# Supplementary material for: Immune evasion and membrane fusion of SARS-CoV-2 XBB subvariants EG.5.1 and XBB.2.3
Source: Emerg Microbes Infect. 2023 Oct 11;12(2):2270069. doi: 10.1080/22221751.2023.2270069 (PMC10606793; doi:10.1080/22221751.2023.2270069)
Supplement: Supplemental Material [file TEMI_A_2270069_SM6979.pdf]

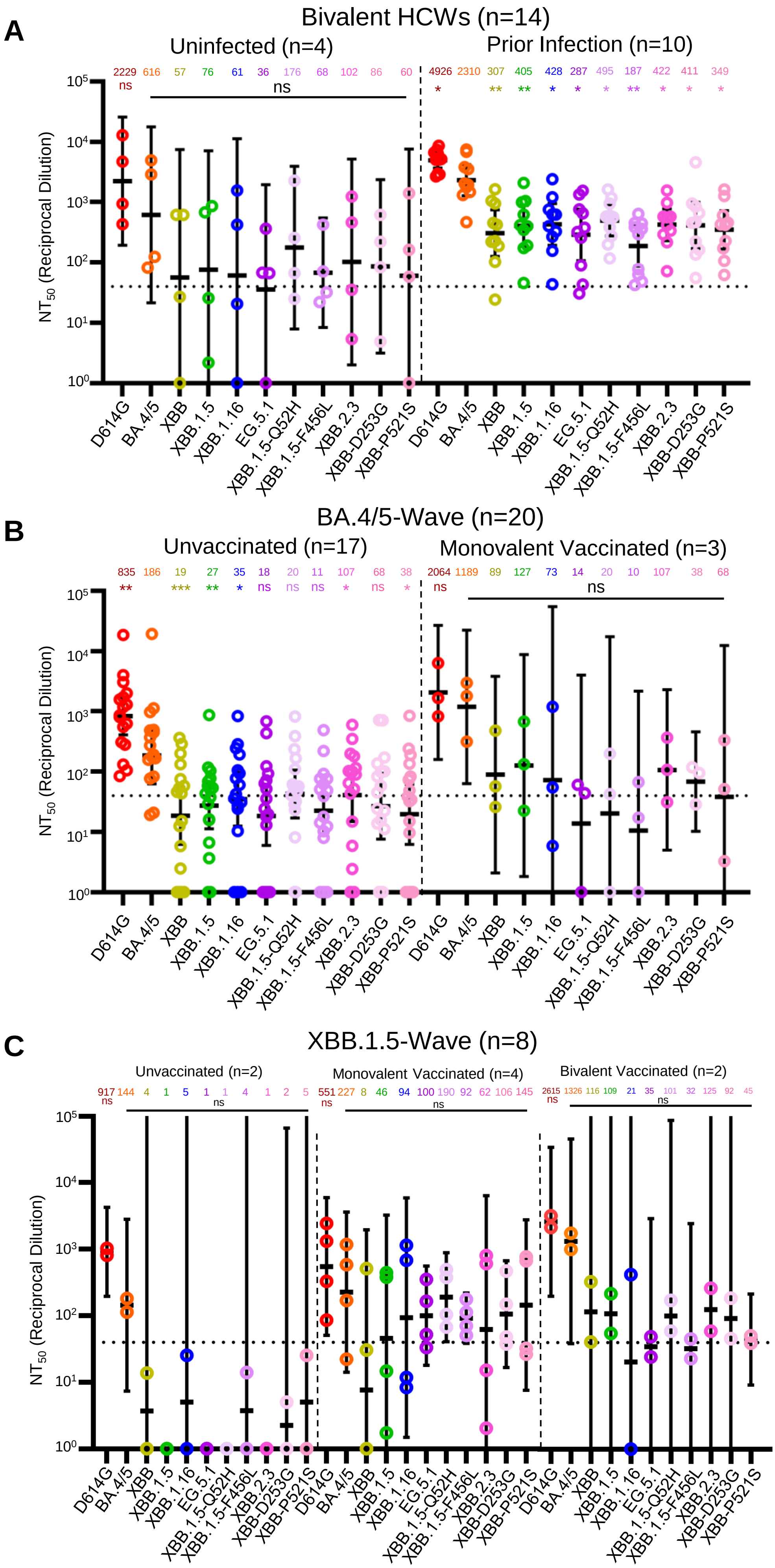

A
Bivalent HCWs (n=14)
Uninfected (n=4)
Prior Infection (n=10)
NT50 (Reciprocal Dilution)
B
BA.4/5-Wave (n=20)
Unvaccinated (n=17)
Monovalent Vaccinated (n=3)
C
XBB.1.5-Wave (n=8)
Unvaccinated (n=2)
Monovalent Vaccinated (n=4)
Bivalent Vaccinated (n=2)
D614G
BA.4/5
XBB
XBB.1.5
XBB.1.16
EG.5.1
XBB.1.5-Q52H
XBB.1.5-F456L
XBB.2.3
XBB-D253G
XBB-P521S

Figure S1

Figure S1: Subgroup analyses of infected and vaccinated individuals in neutralization data cohorts (Related to Figure 2). Pseudotyped lentivirus bearing spikes of interest was used for a neutralization assay to determine neutralization titers for **(A)** bivalent mRNA vaccinated HCWs (n=14), **(B)** first responders and household contacts infected during the BA.4/5 wave in Columbus, OH (n=20), and **(C)** first responders infected during the XBB.1.5 wave in Columbus, OH (n=8). **(A)** The bivalent cohort was split into individuals who had no incidence of breakthrough infection before sample collection (n=4) and those that did experience breakthrough infection (n=10). **(B)** The BA.4/5-wave cohort was split into unvaccinated individuals (n=17) and individuals that received 3 doses of monovalent mRNA vaccine (n=3). **(C)** The cohort was divided into unvaccinated individuals (n=2), people that received 2 or 3 doses of monovalent vaccine (n=4), and people who received at least 3 doses of monovalent vaccine and a bivalent booster (n=2). Plots depict geometric mean neutralization tiers above each variant. The horizontal dashed line at NT50 = 40 represents the limit of detection for the assay. Significance was determined using a repeated measures one-way ANOVA with Bonferroni post-test within each group. Log10 transformed neutralization titers were used to determine significance throughout. p values are displayed as $*p < 0.05$, $**p < 0.01$, $***p < 0.001$, and ns $p > 0.05$.
